# Commentary: Thiamine Deficiency in Tropical Pediatrics: New Insights into a Neglected but Vital Metabolic Challenge

**DOI:** 10.3389/fnut.2016.00058

**Published:** 2017-01-16

**Authors:** Heitor Pons Leite, Lúcio Flávio Peixoto de Lima, Tulio Konstantyner

**Affiliations:** ^1^Pediatrics, Discipline of Nutrition and Metabolism, Federal University of São Paulo, São Paulo, Brazil; ^2^Pediatrics, Pediatric Intensive Care Unit, Federal University of São Paulo, São Paulo, Brazil

**Keywords:** thiamine deficiency, refeeding syndrome, malnutrition, beriberi, intensive care units, pediatric

Although there have been health-care improvements in recent decades, it is a fact that nutritional deficiencies persist in children worldwide (1, 2). One of these is thiamine deficiency (TD). In a comprehensive review, Hiffler et al. (3) have addressed two relevant questions for the management of TD in resource-poor settings that are as follows: (1) the early diagnosis of TD depends on clinical awareness and (2) the need for early thiamine supplementation during the refeeding of malnourished children. The review adds significant insight into the problem and gives the opportunity to discuss diagnosis, management, and the rationale for including thiamine in children nutritional support.

Thiamine deficiency is less common than in the past but still occurs endemically as beriberi in children and nursing mothers living in rural areas of Southeast Asia (4–9) and as outbreaks in different parts of the world (10–12). The clinical spectrum of TD may vary according to the clinical setting, age, and individual susceptibility (13). Infantile beriberi typically presents as heart failure, dysphonia, and increased irritability (5, 14, 15). However, the classical diagnostic criteria for infantile *beriberi* may be non-specific for diagnosis of TD in endemic regions, where the disease may often be clinically unapparent (5). As early symptoms are non-specific, biochemical analysis is unavailable in resource-limited settings, and food intake records are usually inaccurate for a timely diagnosis of deficiency, it is not known just how widespread subclinical TD is.

An issue not addressed in this review is that the clinical expression of a micronutrient deficiency is a result of a progressive process whose duration is variable, dependent on reserves, consumption, and food intake. In an initial phase, there is a depletion of the reserves followed by decreased intracellular concentration. If inadequate intake persists, metabolic changes occur, followed by non-specific functional defects (subclinical deficiency). The end result of this process will be a clinical deficiency state and ultimately death (16). Given its very limited body stores, the time taken to pass through these different phases are shorter for thiamine (13). This can be particularly worrying in the case of malnourished and critically ill patients.

A comment should be made on the laboratory diagnosis of TD. Hiffler et al. refer to the limitations of serum or whole blood thiamine as a marker of thiamine status during the systemic inflammatory response. This is true regarding serum or plasma thiamine (which is transiently reduced following an inflammatory insult), but not for whole blood thiamine. The erythrocytes contain approximately 80% of the total thiamine in whole blood, predominantly thiamine diphosphate (TDP). TDP concentration in erythrocytes is a good indicator of body stores and correlates strongly with that in whole blood (17). Both whole blood and red cell TDP concentrations, as assessed by high-performance liquid chromatography, are accurate indicators of thiamine status. In addition, direct measurements of thiamine stores are better than functional assays (i.e., erythrocyte transketolase activity) because the latter can be influenced by factors other than vitamin deficiency (17, 18).

The risk of TD in the ICU setting is another concern, especially in patients in whom parenteral nutrition or full-rate enteral tube feeding is delayed because of fluid restriction, hemodynamic instability, or gastrointestinal intolerance. Thiamine supplementation is recommended for these patients and those with heart failure, receiving diuretics, or renal replacement therapy (19, 20). The use of thiamine as a potential metabolic resuscitator has been tested in septic shock patients. In a randomized controlled trial by Donnino et al., thiamine administration did not improve lactate levels or clinical outcomes in adults with septic shock the overall group. However, it is noteworthy that in those with baseline TD, patients in the thiamine-supplemented group had lower serum lactate at 24 h and in-hospital mortality (21). Further studies are necessary to determine which groups of patients are expected to benefit from this intervention.

Apart from being associated with increased risk of illness-related mortality in sick children in poor areas (22), TD may cause long-term sequelae, and not only in developing countries. In Israel, two deaths from cardiomyopathy, neurological deficits, and developmental delay were documented in infants exclusively fed soy formula that did not contain thiamine (23).

Thiamine supplementation at initiation of severe malnutrition treatment is not the current practice in pediatric hospitals or ambulatory settings. Furthermore, thiamine content of milk formulas given to malnourished children is not enough to satisfy their estimated requirements during the refeeding, as depicted in Table 1 of the review (3). Since children with severe malnutrition may have low or borderline thiamine stores, they are prone to develop TD during refeeding without thiamine supplementation. Given that beriberi clinical features are non-specific for TD diagnosis, a policy recommendation for empirical therapy with thiamine would be justified for sick children living in endemic areas, emergency-affected populations, and those patients who are critically ill and at risk of TD (4, 24–26). Laboratory confirmation is not required to treat at-risk patients in low resource settings. No tolerable upper intake level has been set for thiamine (27). In severe deficiency states, oral and intravenous administration of doses far above the daily recommended intake for healthy people is considered safe.

To date, TD continues to be under-recognized in malnourished children. The review by Hiffler et al. calls to attention the need for thiamine supplementation in critically ill patients and reminds us that TD must be regarded as a public health problem in poor areas and humanitarian fields. Clinical awareness and early thiamine supplementation are the safest approaches to prevent deficiency-related complications and mortality.

## Author Contributions

HL conceived, wrote, and revised the manuscript. TK and LL contributed to the writing and critically revised the manuscript. All the authors gave their final approval.

## Conflict of Interest Statement

The authors declare that the research was conducted in the absence of any commercial or financial relationships that could be construed as a potential conflict of interest.
